# Some Remarks on the Angustura Bark

**Published:** 1792

**Authors:** George Wilkinson

**Affiliations:** *Surgeon at Sunderland, Member of the Royal College of Surgeons and Honorary Member of the Chirurgo-Physical Society of Edinburgh,* &c.


VII. *Some Remarks on the Angustura Bark. Communicated in a Letter to Dr.* Simmons *by Mr.* George Wilkinson, *Surgeon at Sunderland, Member of the Royal College of Surgeons and honorary Member of the Chirurgo-physical Society of Edinburgh,* &c.

MORE than a year having elapsed since the date of my former letter * to you on the Angustura bark, and the numerous trials I have since made of it, together with the liberal communications of several of my medical friends, having enabled me to speak more decisively of its effects, I take the liberty of communicating to you the following additional remarks on the subject:

These remarks would have been more extended, had I not been anticipated in much of what I might otherwise have said, particularly with respect to the chemical history of this drug, by Mr. Brande, in his valuable essay on the Angustura bark lately published.

I had made several experiments to ascertain the properties of this bark before Mr. Brande's

* See the London Medical Journal, Vol. XI. p. 331.

work work was announced, and I have ſince repeated many of thoſe he has deſcribed; but as my reſults have been nearly ſimilar to his, it ſeems unneceſſary to relate them. I ſhall, therefore, content myſelf with collecting from my notes ſuch detached facts and obſervations relative to this new remedy as may ſerve ſtill farther to aſcertain its properties, its effects in different diſeaſes, and the doſes and modes of exhibition in which it ſucceeds the beſt.

The taſte of this bark, which is a powerful bitter, has been compared, by many to whom I have ſhewn it, to Columbo root, to camomile, and to Caſcarilla bark; yet in its chemical properties it differs in many reſpects from either of thoſe ſubſtances. In its ſenſible qualities it may be ſaid, in ſome degree, to reſemble the Peruvian bark; though, from experiments, it would ſeem to be ſuperior to it in its antiſeptic powers.

I have been at much pains to render the Anguſtura bark more agreeable to the palate by covering its taſte with different aromatics, ſuch as lemon and Seville orange peel, cloves, cardamoms, nutmegs, compound tincture of lavender, and cinnamon. The two firſt may be uſed with it, to great advantage in this reſpect, in infuſion; cloves or cardamoms ſeem only to add to its pungency ; while the three latter, particularly cinnamon, render it more grateful to the ſtomachs of moſt patients.

The mildeſt form in which it can be adminiſtered is that of a cold infuſion ; next to this follows the warm infuſion ; but ſo partially does it give out its properties in theſe forms, that the ſame bark boiled (though infuſed before) in a pint of water to two thirds makes a decoction much ſuperior in ſtrength to either of the infuſions.

Alkaline and neutral ſalts render this bark leſs agreeable to the palate.

Acids, particularly the vitriolic, by precipitating its gummy and reſinous parts, diminiſh its pungency. Thus combined it may be better adapted to ſome ſtomachs ; and reſembles the camomile infuſion when united with the vitriolic acid.

With Port, Liſbon, and moſt other wines, if we except Mountain, Frontiniac, and Raiſin, this bark is not very pleaſant ; the three latter, but particularly raiſin wine, have in general been found to be moſt agreeable. Particular inſtances will ſometimes, though not often, occur where wine in general may diſagree : in all caſes, however, I have found it much better to dilute dilute the wine with an equal proportion of water.

In fome cafes, as in diarrhœa, dyfentery, &c., where the ftomach and inteftines are extremely irritable, fo as to eject this and other medicines, it may require a fuitable addition of opium; but in general it will be found to agree beft in the fimpleft form, and to fit much better on the ftomach than the Peruvian bark, or any other bitter, provided it be given in a proper dofe, which even in adults I have feldom increafed to more than thirty grains, commonly beginning with from ten to twenty grains of it at a dofe.

What has been obferved refpecting the fimple mode of adminiftering it has been confirmed to me by Dr. Collingwood, of this neighbourhood, who, in moft of his trials of this bark, has exhibited it in powder, and that in the moft fimple form.

The good effects I had formerly experienced from the ufe of this bark in two cafes of intermittents *, encouraged me to try it in others that have fince occurred.

* London Medical Journal, Vol. XI. page 336.

The firſt of theſe took place in September, 1790; the ſubject of it was a man, thirty two years of age, who was ſeized with the uſual ſymptoms of fever, accompanied with head-ach, ſlight ſickneſs, and coſtiveneſs. Antimonials were given, in ſmall doſes, at proper intervals, and at the end of about twenty hours his fever went off. On the third day he was attacked with ſhivering, ſucceeded by the uſual ſymptoms of an intermittent. An emetic was now preſcribed: his coſtiveneſs was removed; and he took the Peruvian bark, in ſubſtance, in doſes of two ſcruples, every three hours, mixed with two or three table-ſpoonfuls of a decoction of the ſame. He had one fit afterwards, having then taken three ounces of the bark in powder, and nearly the ſame quantity of it in decoction. The uſe of it was continued, in leſs frequent doſes, for a fortnight, and he then thought himſelf recovered. At the end of the third week, however, he was again ſeized with a rigor and hot fit. The Anguſtura bark was now adminiſtered, in powder, in doſes of from twelve to fifteen grains, in a ſmall quantity of raiſin wine and water, at the ſame periods as the Peruvian bark had been. He never had another fit afterwards, and the whole of the quantity quantity given was juſt three drachms and a half.

To another patient who fell under my management, and whoſe caſe was alſo a tertian, nearly ſimilar, though leſs violent than the laſt-mentioned one, no other medicines were uſed excepting ſome mild antimonials, and a gentle purge given previouſly to the uſe of the Anguſtura bark, of which he took half an ounce, in powder, in doſes of from twelve to fifteen grains: he had three fits prior to his taking it, but afterwards remained quite well.

In a third caſe of a tertian, the ſubject of which was a female, twenty-eight years of age, this bark proved leſs efficacious; for though it ſeemed to put a ſtop to the fits after having been taken, in ſubſtance, to the amount of a dozen doſes of twelve grains each, yet notwithſtanding the quantity was increaſed to three grains more, the fits again recurred, and the medicine was laid aſide after ſhe had taken ſix drachms of it. This patient, who was ſubject to obſtinate coſtiveneſs, (on account of which a purgative medicine was adminiſtered previouſly to the uſe of the bark) had impaired her health by the too frequent uſe of ſpirits, and took the bark in a very irregular manner, ſo that that it could hardly be ſaid to have had a fair trial. The fits afterwards yielded to the uſe of the arſenical ſolution, but her general health was not till ſome time after reſtored.

Mr. Akenhead, ſurgeon, of this town, has communicated to me the following account of the good effects of this bark in a tertian ague:

"Some time in July laſt a robuſt man, thir-

" ty-ſix years of age, in conſequence of expo-

" ſure to cold from getting wet, was attacked

" with an intermittent. Before I ſaw him he

" had been ill for ſeveral days, and had had

" three or four fits, which recurred every other

" day. After having adminiſtered an emetic

" of ipecacuanha, I was induced to make trial

" of the Anguſtura bark, which I gave him

" in decoction with a ſmall quantity of com-

" pound tincture of lavender, and in doſes of

" three table-ſpoonfuls every three hours. He

" had but one fit afterwards, and was perfectly

" cured; having taken in the whole only a pint

" and a half of the decoction."

Repeated experience alone can enable us to decide with preciſion on the comparative effects of the Anguſtura and Peruvian barks in fevers I have not had many opportunities of this ſort

ſince I became acquainted with the Anguſtura bark; but in three caſes of typhus that have come under my obſervation, in the beginning of this year, I can truly ſay, the effects of this bark have by no means fallen ſhort of the Peruvian. One of them was the more remarkable, as the patient at the time of my uſing it was in a very hopeleſs ſtate. His age was ſixty-four years, his occupation ſedentary, and he was of a ſtudious turn of mind. For two or three days prior to my viſiting him he had laboured under languor, decreaſe of appetite, ſlight thirſt, nauſea, and head-ach. His pulſe, when I firſt ſaw him, was quick, feeble, and ſomewhat irregular; his tongue foul, his ſkin dry, and his belly coſtive. A gentle laxative was at firſt exhibited, and after this mild antimonials, but with no apparent benefit. Wine and other cordials were next had recourſe to, and by theſe he ſeemed to be ſomewhat relieved till about the ninth or tenth day, when he was ſeized with a diarrhœa. Opiates and other remedies were now adminiſtered, but to no purpoſe. His ſkin felt cold to the touch; his pulſe ſunk, and his ſtrength ſeemed to be nearly exhauſted. In this alarming ſtate I reſolved

ſolved to try the effects of the Anguſtura bark. The following form was uſed :

℞. Infuſ.* cort. Anguſturæ ℥vj.

Tincturæ * ejuſdem ℥ſs.

Pulv. ——— ℈j.

Tincturæ opii guttas xx.

——— lavend. comp. gutt. xl. M.

Of this mixture three table-ſpoonfuls were given every four hours. The firſt doſe gave him great relief, and the next day I found his diarrhœa quite gone, his ſkin warm and moiſt, and his deſire for food returning. He took a proper ſupply of nouriſhment, and at the end of four days was capable of ſitting up ; the mixture was continued, and he very ſoon regained his health.

In the two other caſes, the ſubjects of which were females, the ſymptoms of fever were more ſtrongly marked ; the general plan I adopted in theſe conſiſted in the exhibition of antimonials, at firſt, in a doſe ſufficient to excite vomiting, and afterwards in ſmaller doſes as relaxants, with a gentle laxative occaſionally. To one of theſe patients the Anguſtura bark, in powder, was given, in doſes of fifteen grains

* See London Medical Journal, Vol. XI. page 333.

in in a glafs of white wine and water, every four hours after the firft intermiffion, or rather remiffion, that I could perceive, and which happened on the fourth day. The fame remedy was afterwards continued, and the patient recovered in a few days. In the other cafe, which was treated nearly in a fimilar manner, at the beginning, as the former, a remiffion took place on the third day, and the decoction of Anguftura bark being then given, put a ftop to the difeafe.

Four cafes of fever in lying-in women have occurred to my notice. All of thefe affumed the form of remittents or quotidians, and in all of them this bark was ufed with great fuccefs. It was chiefly given in infufion or decoction, combined with the faline mixture, during the remiffions, and fometimes accompanied with the tincture and a fmall quantity of the powder. In each of thefe cafes the fymptoms of fever began to fubfide foon after I had recourfe to this remedy, and its ufe was continued till a complete recovery was effected.

One of thefe patients, and the firft that fell under my management, was my fifter, a perfon of an irritable habit, who had much fever, accompanied with delirium. She had never, at any time, been able to relifh the Peruvian bark, but took this very readily and without inconvenience.

Another patient, with whofe ftomach this bark difagreed, by inducing an uneafy fenfation of heat, took it afterwards by combining with its infufion a portion of magnefia.

In diarrhœas this bark, fo far as I can judge from my own trials of it, feems to ftand unrivalled by any other medicine of the fame clafs that has hitherto been difcovered.

I could relate feveral cafes of recent as well as chronic diarrhœa in proof of this, were not the fact already fo fatisfactorily eftablifhed by the inftances given by Mr. Brande from his own experience, and that of Dr. Willan, of its efficacy in complaints of this kind. I flatter myfelf, however, that the infertion of the two following cafes, communicated to me by two refpectable medical friends, will not be unacceptable to you.

The firft of thefe is from Mr. Salkeld, furgeon at Durham. The patient was a child, four months old, who had a diarrhœa, of feveral days ftanding, accompanied with gripes and bloody ftools. About four grains of the Anguftura bark, in powder, were directed to be given, given, at proper intervals, in a ſpoonful of cinnamon water. After the ſecond doſe the appearance of blood in the ſtools ceaſed, and in about two days, by perſevering in the uſe of the medicine, the complaint was completely removed.

For the other caſe I am indebted to Dr. Collingwood, whoſe account of it is as follows:

" A married woman, twenty-five years of

" age, was attacked, three weeks after delivery,

" with a diarrhœa. Various remedies of the

" tonic and aſtringent claſs were employed,

" from ſome of which ſhe experienced good

" effects, but none of them afforded her per-

" manent relief. She continued in this ſtate,

" better and worſe, till ſix months had elapſed;

" and as the diſeaſe ſtill remained, I adviſed

" her to wean the child: this was complied

" with, but no good was produced. To re-

" peat the variety of means, both of medicine

" and diet, that were adopted, would be tedi-

" ous. Every thing ſeemed in vain; for, after

" a period of ten months from its commence-

" ment, the complaint had reduced her to an

" extreme extenuation and debility of body;

" her appetite was depraved; her eyes appear-

" ed ſunk; her complexion was quite ſallow;

" her pulſe quick and weak; and her ſtools

" frequently amounted to the number of

" twelve, fourteen, and latterly to twenty or

" more, in the ſpace of twenty-four hours.

" When you gave me ſome of the Anguſtura

" bark, I muſt confeſs that, before I tried it, I

" deſpaired of its ſucceeding in this caſe. The

" event, however, agreeably diſappointed me.

" I began by the exhibition of an emetic of

" ipecacuanha, ſeveral of which had been ad-

" miniſtered before. She then took ten grains

" of Anguſtura bark, in powder, in rice water,

" after every ſtool. The very firſt doſe re-

" lieved her, and when ſhe had taken five the

" diarrhœa ſeemed to ceaſe, and ſhe recovered

" by the repetition of a few more doſes, the

" whole of which amounted to a dozen. Her

" appetite and digeſtion ſoon improved, and at

" the end of three weeks ſhe was perfectly

" cured."

In that ſpecies of diarrhœa occaſioned by the quick abſorption of the adeps in children, and which is frequently the conſequence of dentition, though it ſometimes proceeds from other cauſes, ſuch as obſtruction of the meſenteric glands, &c., this bark has proved uſeful, particularly in caſes where the complaint ſeemed to depend on a ſtate of morbid irritability connected with teething. In moſt of theſe caſes there is more or leſs of pyrexia, accompanied with depraved appetite, thirſt, nauſea, and ſometimes vomiting. I have, therefore, generally adminiſtered a mild emetic of ipecacuanha previouſly to the uſe of the Anguſtura bark in ſuch caſes; and where there was reaſon to aſcribe the complaint to viſceral obſtruction I have given, during its uſe, occaſional doſes of calomel.

And here I may obſerve, that this bark is, for ſeveral reaſons, preferable to the Peruvian as a medicine for children: they take it much more readily than they do the latter; and it is leſs apt to diſagree with the ſtomach and run off by the inteſtines, which the Peruvian bark is too apt to do from the large doſes that are neceſſary to produce a deciſive effect.

In the true dyſentery I have had an opportunity of trying it only in one caſe, of a patient who had taken a variety of remedies without any permanent good effect. At the time I firſt ſaw him he had a conſiderable degree of pyrexia, with frequent gripes, and mucous ſtools intermixed with more or leſs of blood. By the uſe of the Anguſtura bark his gripes and evacuations ſoon ceaſed; his appetite returned;

 the the ſymptoms of fever left him, and he gradually recovered.

The frequent failure of the Peruvian bark (particularly when given in ſubſtance or decoction) in dyſpepſia, has induced many practitioners to combine it, in ſuch caſes, with a number of other ingredients of the bitter claſs; and among thoſe we may enumerate the late Profeſſor Whytt, of Edinburgh, who was often under the neceſſity of laying it aſide, and depending on that efficacious and elegant preparation, his tincture, or on the warmer bitters alone *. In caſes of this kind the Anguſtura bark will be found to have great efficacy, as I can aſſert from my own experience, having adminiſtered it with ſucceſs in numerous inſtances where the Peruvian bark, and other medicines of the bitter claſs, had failed.

The teſtimony of ſeveral of my medical friends, who have tried it in ſuch caſes, is alſo ſtrongly in its favour: my friend Mr. Akenhead, for inſtance, obſerves to me, that "he "has given it a trial in ſeveral caſes of indi- "geſtion with the beſt effects; and that al-

* Obſervations on nervous Diſorders. 8vo. Edin. 1765. pages 328 and 372.

" though he has long been in the habit of ta-

" king the Peruvian bark, and other bitters,

" for a ſimilar complaint, to which he is often

" ſubject, yet from the benefit he has lately

" derived from the Anguſtura bark, and the

" eaſe with which it ſits on his ſtomach, he

" cannot but give it the preference :" and from Mr. Salkeld I learn, that " in a caſe of dyſ-

" pepſia, from an immoderate uſe of ſpirits,

" in which the Peruvian and Quaſſia barks, Co-

" lombo root, and other remedies, had been

" tried during at leaſt three months, without

" the leaſt advantage, the patient experienced

" almoſt immediate relief from fifteen grains

" of the Anguſtura bark adminiſtered twice a

" day." In this caſe the doſe was afterwards increaſed to twenty-five grains, which ſat eaſy on the ſtomach, and by perſevering in the uſe of the medicine the patient gradually recovered.

Theſe effects of the Anguſtura bark confirm the opinion entertained of it by Dr. G. Pearſon, who, though unwilling to allow it equal efficacy with the Peruvian bark in the cure of intermittents, conſiders it " as a medicine that

" will produce the effects of the warm vege-

" table bitters, eſpecially of the camomile, but

"with more efficacy, and more agreeably to

"the ſtomach; and that it will conſequently

"render all the other articles under the head

"of *amara calida* unneceſſary *."

In caſes of debility, or increaſed irritability, whether apparently proceeding from a diminiſhed tone of the ſtomach, or from local affections of certain parts, the Anguſtura bark has appeared to me to produce effects ſimilar to thoſe of the Peruvian bark, but with theſe advantages, that the effects were more immediate and ſudden, and the doſes much ſmaller than thoſe uſually given of the latter bark. Hence I have employed it with much ſatisfaction in ſcrophulous affections; in violent accidents producing inflammation, ſymptomatic fever, weakening diſcharges, &c.; and after ſome of the greater operations of ſurgery.

In theſe caſes I have ſometimes alternately adminiſtered both this and the Peruvian bark to the ſame patient. To me the evidence has conſtantly appeared to be in favour of the Anguſtura; and every patient who took it, whe-

* See a letter from Dr. Pearſon to Mr. Brande, annexed by the latter to his Experiments and Obſervations on the Anguſtura Bark.

ther ther firſt or laſt uſed, preferred it, aſſuring me hey found from it more benefit.

In certain complaints, more eſpecially venereal caſes, where the indiſcriminate and oftentimes improper uſe of mercury ſeems to produce more dreadful effects than even the diſeaſe itſelf, I have given this bark with great advantage.

A gentleman who laboured under a lues venerea, though not accompanied with ſecondary ſymptoms, had rubbed in and taken conſiderable quantities of mercury for the ſpace of about two months. When I firſt ſaw him he appeared greatly emaciated; had an ulcerated mouth, with profuſe ſpitting, hectic heat, thirſt, loſs of appetite, and diarrhœa, accompanied with griping. He had then been for ſome time taking opium and Peruvian bark, but without any apparent benefit; for he got little or no ſleep, and the ulcers in his groin were very painful, and looked unpromiſing.

Laying aſide his other medicines, I adviſed him to take, at bed time, ten grains of the Anguſtura bark, in powder, in a little raiſin wine and water, which doſe was repeated towards morning. At the ſame time I recommended to him a more generous diet than he had of late

 been been accuſtomed to, together with a moderate uſe of wine. The good effects of this mode of treatment were ſoon conſpicuous; for the very next day he began to have a return of appetite; his diarrhœa and other unfavourable ſymptoms ſoon began to leave him; the ulcers took on an healing diſpoſition; his ſtrength of body returned very faſt; and he gradually recovered.

Mr. Salkeld has mentioned to me the effects of this bark in a caſe of hooping cough. To a girl, ſix years of age, who had been affected with this diſorder ſo much as to produce conſiderable hæmorrhages from the noſe and ears, after having preſcribed for a month, without effect, a mucilaginous mixture of emetic tartar and oxymel of ſquills, ſo as to keep up a gentle nauſea, he ordered an infuſion of the Anguſtura bark, to four ounces of which were added thirty drops of the tincture of cantharides. Of this mixture a table-ſpoonful was directed to be taken three or four times a day. After taking a few doſes the child was much better, and, to uſe her mother's words to Mr. Salkeld, "hardly ever coughed afterwards." The medicine was continued, and the child ſoon got quite well.

Mr. Salkeld has found it very efficacious in a cafe of fcrophulous ophthalmia, which had refifted the ufe of blifters, leeches, and a variety of other remedies, external and internal. A decoction of the Anguftura bark was applied as a wafh, and cloths dipped in it were kept upon the eyes at night. In twenty-four hours the patient was able to look up, and bear the light, which he had not been able to do for near a month before. The application was continued, and he foon got well.

I myfelf have fince tried it in a girl, twelve years of age, who had a fimilar complaint, from the fame caufe, in both eyes. At the time I firft faw her fhe had been almoft blind for five weeks; I directed her to apply the infufion to her eyes in the fame manner Mr. Salkeld's patient had done, and in three days, without the ufe of any other remedy, fhe was able to bear the light, and in a fhort time was perfectly cured. To this account of its effects, as a topical application, I am able to add, that it has been ufed as a fomentation in a cafe of partial gangrene produced by an accident, and with evident advantage, as it foon corrected the fœtor.

In ill-conditioned ulcers, alſo, the powder of this bark has been found of excellent uſe, by acting as a mild eſcharotic ; and a ſtrong decoction of it, applied as a waſh, to ſores of this deſcription, has been found of conſiderable ſervice.

Before I conclude this letter I muſt beg leave to obſerve to you, that in every caſe in which I have adminiſtered this bark I have been anxious to give it a fair trial ; and with this view have never combined it with any other powerful remedy, or with ſubſtances that could at all influence its effects, except by rendering it perhaps more agreeable to the ſtomach. The reſults of my own experience of its effects are faithfully related ; the communications concerning it, with which I have been favoured by my medical friends, who are men of great accuracy and candour, are given nearly in their own words ; and I truſt that the trials of others will tend to confirm what has been advanced relative to this new remedy, which I cannot but conſider as a valuable addition to the Materia Medica.

*Sunderland,*

October 4, 1791.

VIII. *An*

